# A Group-Enriched Viscoelastic Model for High-Damping Vitrimers with Many Dangling Chains

**DOI:** 10.3390/ma17205062

**Published:** 2024-10-17

**Authors:** Yan Li, Haibo Feng, Jing Xiong, Li Li

**Affiliations:** State Key Laboratory of Intelligent Manufacturing Equipment and Technology, School of Mechanical Science and Engineering, Huazhong University of Science and Technology, Wuhan 430074, China

**Keywords:** damping material, loss modulus, group effect, viscoelastic model, vitrimer

## Abstract

Classical viscoelastic models usually only consider the motion of chain segments and the motion of the entire molecular chain; therefore, they will cause inevitable errors when modeling self-healing vitrimer materials with many group movements. In this paper, a group-enriched viscoelastic model is proposed for self-healing vitrimers where the group effect cannot be neglected. We synthesize a specific damping vitrimer with many dangling chains, surpassing the limited loss modulus of conventional engineering materials. Due to the dangling chains, the damping capability can be improved and the group effect cannot be neglected in the synthesized damping vitrimer. The group-enriched viscoelastic model accurately captures the experimental damping behavior of the synthesized damping vitrimer. Our results indicate that the group-enriched viscoelastic model can improve the accuracy of classical viscoelastic models. It is shown that the group effect can be ignored at low frequencies since the chain segments have sufficient time for extensive realignment; however, the group effect can become significant in the case of high frequency or low temperature.

## 1. Introduction

Mechanical systems often generate a variety of vibrations and noise during operation. These dynamic responses affect the operational stability and service life of the equipment [1,2]. Especially in fields such as aerospace, rapid transit, and high-precision instruments, vibrations can have serious consequences [3,4]. To address these issues, an increasing number of engineers and researchers are focusing on the application of damping materials [5,6,7]. Damping materials can effectively absorb and dissipate mechanical vibrations, thereby reducing the amplitude of vibrations and noise levels [8]. By introducing appropriate damping materials into mechanical systems, it is possible to significantly enhance the stability and durability of the equipment [9]. Therefore, the proper selection and application of damping materials are crucial for improving the dynamic response of mechanical systems and extending the service life of equipment [10,11,12]. Polymer materials possess excellent mechanical properties, chemical resistance, and other advantageous characteristics, and have garnered significant attention over the past few decades [13]. The viscoelastic properties of polymer materials can be utilized to convert mechanical vibration energy into thermal energy dissipation, thus suppressing vibration and noise [14]. To maximize the vibration and noise reduction effect of damping materials, it is necessary to develop wide temperature domain damping and wide frequency band damping materials with excellent damping performance at a specific temperature and frequency range [15,16].

Vitrimer self-healing material, which has recently gained much attention as a new type of polymer material, has the excellent damping properties of polymers [17,18,19]. Moreover, due to its covalent adaptable network (CAN) [20], this polymer is fluid while maintaining the integrity and insolubility of a crosslinked network at high temperatures. This allows it to be remolded and reprocessed [21,22]. These properties confer self-healing capabilities and recyclability to the material, offering excellent prospects for future development. The formation of CANs has been achieved by mechanisms such as ester exchange [23], disulfides [24], and imine bonds [25,26]. Some of these vitrimers of disulfide bonds do not require catalysts [27] and have relatively superior mechanical properties, which makes the disulfide bond mechanism more widely applicable.

The performance of damping materials is often evaluated using the loss factor and loss modulus. The loss factor indicates the damping performance of the material [28] and the product of the storage modulus (the elastic modulus is used in statics), and the loss factor (such a product is the loss modulus) is commonly viewed as a figure of merit in damping applications [29]. In freely damped structures, the effect of the unconstrained damping layer depends on the loss modulus. Therefore, the development of a viscoelastic model that accurately predicts the loss modulus is essential for the application of damping materials [30,31].

Many researchers have developed various models to investigate the mechanical properties of CAN. Long et al. [32] created the initial three-dimensional continuum mechanics model utilizing a phase evolution modeling technique to represent the shape retention of CANs following a thermoforming process. Ma et al. [33] developed a finite deformation model that incorporates a standard linear solid viscoelastic framework along with photo-viscoelastic elements connected in series. This innovative approach effectively captures the behaviors of stress relaxation and tensile creep. Luo et al. [34] have shown that the bond exchange reaction of CAN can also cause energy dissipation. The coupling effect in the glass transition region improves the damping properties of the material. These studies reveal some of the mechanisms by which materials exhibit excellent properties.

The viscoelastic model is based on the structural properties of the polymer and the internal molecular motion. The molecular motion of polymers is very complex. The moving units include the whole molecular chain, chain segments, links, side groups, branched chains, and all other atoms and groups [35]. At the molecular level, the CANs contain many dangling chains [36]. These free chain structures rub against each other under the action of external forces, which further improves the energy dissipation ability of the material. To simplify the model, usually, only the chain segment motion, as well as the motion of the whole molecular chain, is considered. The motion of macromolecules becomes more pronounced under the influence of temperature and external forces, which will constantly change their configuration and conformation, whereas at lower temperatures or higher frequencies, the macromolecular motion is hindered and the influence of smaller-scale structures on the viscoelastic effect of polymers is improved. Davis et al. [37] established a quantitative relationship between the molecular structure of polymers and their dynamic mechanical properties. In polymers with CANs, internally contained dangling chains make this small-scale vibration more pronounced. Therefore, a viscoelastic constitutive model that takes into account the motions of the groups is more suitable for describing the viscoelastic effects of vitrimers. The constitutive model for this term can be developed based on the contribution of the groups to the loss modulus.

In this work, experiments will be carried out to test the properties of the material. A group-enriched viscoelastic model will be developed. The effect of groups on the viscoelastic behaviors of the vitrimer self-healing material will be revealed by characterizing the group effect at low temperatures and high frequency. The identification of the parameters of the constitutive model is based on the results of Dynamic Mechanical Analyzer (DMA) experiments. The dynamic response of the material is predicted based on the model, and the effect of the group on the viscoelasticity is quantified. This paper is organized as follows: Section 2 describes the preparation of the experimental materials and the testing of their properties. Section 3 describes the viscoelastic model and the temperature dependence of the materials. In Section 4, the parameters of the group-enriched model are obtained. The modification of the model is verified by comparing the experimental results.

## 2. Viscoelastic Model Incorporating Group Effect

### 2.1. Group-Enriched Viscoelastic Constitutive Model

For the linear deformation case, the movement of the molecular chain dominates the energy dissipation in polymers. However, the dangling chains in self-healing vitrimer materials provide an additional damping effect. Regarding the damping of vitrimers, it can be decomposed into viscoelastic damping and group-driven damping. This is because viscoelastic damping usually only considers the movement of chain segments and the movement of the entire molecular chain, so the group-driven damping characteristics cannot be considered. Thus, the strain energy W(t) considering the memory effect for viscoelastic and group-driven damping can be written as [16]:

(1) $$W\left(t\right)=\frac{1}{2}\int_{-\infty }^{t}\int_{-\infty }^{t}\left[M\left(2t-s_{1}-s_{2}\right)+G\left(2t-s_{1}-s_{2}\right)\right]\frac{\partial \varepsilon }{\partial s_{1}}\frac{\partial \varepsilon }{\partial s_{2}}ds_{1}ds_{2}$$

where *t* is the time; $M\left(2t-s_{1}-s_{2}\right)$ and $G\left(2t-s_{1}-s_{2}\right)$ denote the time-nonlocal kernel functions of viscoelastic damping and group-driven damping, respectively; and $s_{1}$ and $s_{2}$ are the integral variables. Note that for simplicity, we consider ε to be the strain for a one-dimensional problem.

According to Li et al. [38], there is a certain correlation between the intrinsic characteristic length of the material and its intrinsic characteristic time; the smaller the intrinsic characteristic length, the shorter the corresponding intrinsic characteristic time. Then, if only viscoelastic damping is considered, this will cause the damping characteristics in the high-frequency (low-temperature) region to be inconsistent with the experimental results. For this reason, group-driven damping must be considered to achieve accurate characterization of damping characteristics in a wide frequency domain or a wide temperature domain. Thus, the group-enriched strain energy (1) not only provides the viscoelastic kernel function *M*, but also introduces the group kernel function G.

Using the second law of thermodynamics, the constitutive law of the vitrimer can be determined to be in the following form [39]:

(2) $$\sigma =\frac{\partial W(t)}{\partial \varepsilon }$$

where σ denotes the stress. Substituting the strain energy (1) into the constitutive law (2), the stress σ can be expressed as a function of the applied strain ε:

(3) $$\sigma =\int_{-\infty }^{t}\left[M\left(t-\tau_{1}\right)+G\left(t-\tau_{1}\right)\right]\frac{\partial \varepsilon }{\partial \tau_{1}}d\tau_{1}$$

where $\tau_{1}$ denotes the integral variable. According to the relaxation time distribution theory, due to the multiplicity of structural units and the complexity of the motion in viscoelastic damping, the mechanical relaxation process of the vitrimer does not have just one relaxation time, but rather, a wide continuous spectrum. In general, the generalized Maxwell model satisfies the situation of chain relaxation. When the group effect is considered, the deformation of polymers at the molecular level can be composed of two parts from a structural aspect.

The first part is the change in the bond length and bond angle within the molecule caused by the movement of groups in the polymer. This ordinary deformation is usually instantaneous and can be simulated with the modulus $E_{G}$ of a pure elastomer. The second part is the viscous flow caused by the slip of the polymer.

**Figure 1:**
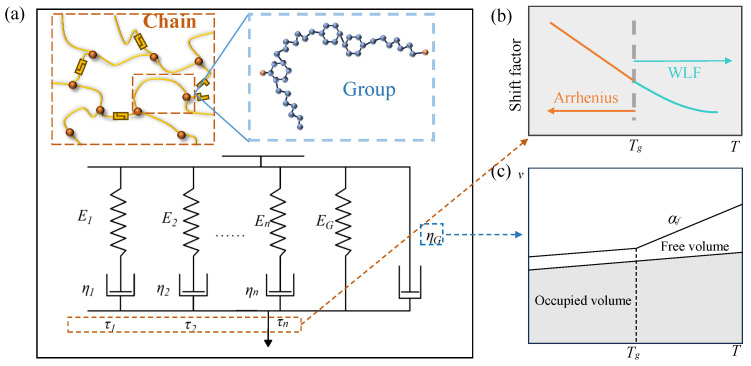
(**a**) Group-enriched model incorporating chain relaxation and group effects. (**b**) Temperature dependence of relaxation time of chain relaxation. (**c**) Specific volume–temperature curves of amorphous polymers.

In the low frequency range, the group effect on the viscous flow is simulated by a dashpot $\eta_{G}$. Under the simplified conditions, the group contribution to the viscoelastic effect results from the simultaneous action of these two parts. Based on this, a group-enriched model was established, as shown in Figure 1a. From a broader perspective, the dashpot that simulates group effects can be seen as a degradation of a Maxwell model. The relaxation time $\tau_{G}$ of the group is close to 0 due to its small size (resulting in very high frequencies). When considering low frequencies of experimental conditions, there exists $\omega \tau_{G}\to 0$ (ω is the angular frequency), and the Maxwell unit degenerates into a dashpot.

**Figure 2:**
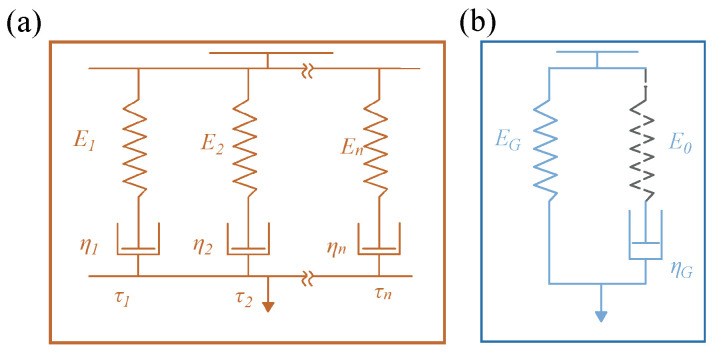
Schematic Diagram of (**a**) the generalized Maxwell model and (**b**) Kelvin–Voigt model.

Differences in the scales of group and chain motions give them different temperature dependencies. The models can be degenerated into the generalized Maxwell model and Kelvin–Voigt model, as shown in Figure 2. The generalized Maxwell model and Kelvin–Voigt model are employed to account for chain relaxation and group effects, respectively. These varying temperature dependencies allow for their independence.

The generalized Maxwell (GM) model contains Maxwell units formed of multiple springs and dashpots, as shown in Figure 2a, and can be used to describe the complex relaxation process of polymers [40,41]. The GM model describes the dynamic mechanical behavior of viscoelastic damping from chain relaxation [42].

Using the concept of the generalized Maxwell model, the time-nonlocal relaxation kernel function *M* in Equation (3) for the viscoelastic damping part can be expressed as [34]

(4) $$M\left(t\right)=\sum_{i=1}^{n}E_{i}\exp\left(-\frac{t}{\tau_{i}}\right)$$

where $E_{i}$ (i=1,2,...,n) is the contribution of the *i*th relaxation process to the viscoelastic elastic modulus, as shown in Figure 1a and Figure 2a, and $\tau_{i}$ is the *i*th viscoelastic relaxation time. The relaxation time $\tau_{i}$ can be expressed as

(5) $$\tau_{i}=\frac{\eta_{i}}{E_{i}}$$

where $\eta_{i}$ is the *i*th viscoelastic dashpot to characterize the viscosity of the *i*th relaxation process.

Using Fourier transform and inverse Fourier transformation, the stress relaxation kernel function *M* can be converted into the complex modulus $M^{*}$. It can be expressed as the storage modulus $M^{\prime }$ and the loss modulus $M^{\prime \prime }$ [43]:

(6) $$\begin{array}{cc} M^{*} & =M^{\prime }+iM^{\prime \prime } \end{array}$$

where

(7) $$\begin{array}{c} M^{\prime }=\sum_{i=1}^{n}E_{i}\frac{\omega^{2}\tau_{i}^{2}}{1+\omega^{2}\tau_{i}^{2}}\;,\;M^{\prime \prime \prime }=\sum_{i=1}^{n}E_{i}\frac{\omega \tau_{i}}{1+\omega^{2}\tau_{i}^{2}} \end{array}$$

Here, ω is the angular frequency, and i is the imaginary sign. When considering only one branch, the standard linear solid model or the Zener damping model can be recovered from Equation (6). That is, the standard linear solid model only has one relaxation time. Since the structural unit of the vitrimer itself has multiplicity and complexity of movement due to its dangling chains and devisable microstructures, there are many relaxation times in our material.

The complex modulus $M^{*}$ in Equation (6) is viewed as the viscoelastic part, which is commonly used to describe the dynamic mechanical behavior of polymers considering chain relaxation. However, the model does not consider the effects of molecular-level vibrations on smaller scales. Therefore, a phenomenological model of group contribution is the key to predicting polymers with dangling chains [44,45,46].

The Kelvin–Voigt model is used to explain the group effect of the vitrimer, as shown in Figure 2b, which can be regarded as the Maxwell unit degenerating into a dashpot. This means that the corresponding relaxation time of the Maxwell unit is $\tau_{G}\to 0$. Using the concept of the Kelvin–Voigt model, the time-nonlocal relaxation kernel function G in Equation (3) for the group-driven damping part can be given by

(8) $$G\left(t\right)=E_{G}+E_{0}\exp\left(-\frac{t}{\tau_{G}}\right)$$

where $E_{G}$ is the contribution of the group effect to the material modulus, and $\tau_{G}$ is the group-driven relaxation time and converges to 0.

Note again that the relaxation time $\tau_{G}$ of the group is close to 0 due to its small size and correspondingly high frequencies. For $\tau_{G}\to 0$, we obtain $\exp\left(-t/\tau_{G}\right)\to 0$ for long-term problems, and $\exp\left(-t/\tau_{G}\right)$ becomes significant only for very short-term problems (or high-frequency problems). Thus, the contribution of $E_{0}$ to the time-nonlocal relaxation kernel function G is notable when considering high-frequency problems.

Similarly, when the Kelvin–Voigt model (8) is used to simulate the group-driven dynamic mechanical behavior, the group-driven complex modulus $G^{*}$ can be expressed as the group-driven storage modulus $G^{\prime }$ and the group-driven loss modulus: $G^{\prime \prime }$:

(9) $$\begin{array}{c} G^{*}=G^{\prime }+iG^{\prime \prime } \end{array}$$

with

(10) $$\begin{array}{c} G^{\prime }=E_{G}\;,\;G^{\prime \prime }=\omega \eta_{G} \end{array}$$

Here, $\eta_{G}$ is the dashpot to characterize the group-driven viscosity, as illustrated in Figure 2b.

It is worth noting that unlike the GM viscoelastic model, in which we usually calibrate its model parameter values by fitting experimental data, the viscosity of the group effect is not an additional fitting parameter in the group-enriched viscoelastic model. It is predetermined using the free-volume theory and the group contribution from previous studies. This approach enables us to determine the group viscosity independently of the fitting process of the GM viscoelastic model.

Combining the viscoelastic effect (4) and the group effect (8), the total time-nonlocal kernel function $E\left(t\right)$ of the group-enriched viscoelastic model can be expressed as

(11) $$\begin{array}{cc} E\left(t\right) & =E_{G}+E_{0}\exp\left(-\frac{t}{\tau_{G}}\right)+\sum_{i=1}^{n}E_{i}\exp\left(-\frac{t}{\tau_{i}}\right) \end{array}$$

Similarly, the total complex modulus $E^{*}$ of the group-enriched viscoelastic model can be given by

(12) $$\begin{array}{c} E^{*}=E^{\prime }+iE^{\prime \prime } \end{array}$$

where $E^{\prime }$ and $E^{\prime \prime }$ are the material’s energy storage and loss modulus, respectively. They are defined as

(13) $$\begin{array}{c} E^{\prime }=M^{\prime }+G^{\prime }\;,\;E^{\prime \prime }=M^{\prime \prime }+G^{\prime \prime } \end{array}$$

Thus, the loss factor tanδ of the vitrimer material can be defined as

(14) $$\begin{array}{cc} \; & \tan\delta =\frac{E^{\prime \prime }}{E^{\prime }} \end{array}$$

Note that the group-driven modification of the loss modulus $E^{\prime \prime }$ is the focus of attention since it represents the combined performance of material stiffness and damping. Often, the loss modulus is used as a figure of merit of damped structures [30].

### 2.2. Quantification of the Group Effect

For the most part, the motion of molecular chains dominates the viscoelastic effect of polymeric materials. However, at lower temperatures and higher frequencies, the motion of the molecular chains is hindered. The influence of smaller-scale groups on the viscoelastic effect comes to the fore. This group contribution can be reflected by the loss modulus and has been proven to be a molecular characteristic [47,48]. Many researchers [49,50] have quantitatively calculated the contribution of each group in terms of the area under the curve (LA, Loss Area). This study refers to this data for parameter identification of the group contribution model. LA has proven to be a molecular feature in polymers such as simple homopolymers, statistically structured copolymers, and interpenetrating polymer networks (IPNS). LA is determined by the structure of individual chains and obeys the additive relationship of the component polymers. However, for vitrimer materials with complex CANs, large-scale movement of polymer chain segments and backbones occurs in the glass transition region. Under lower-temperature conditions, this slip of long chains and their constituent networks dominates the stress relaxation of the polymer. At the same time, the rearrangement of the backbone conformation leads to the possibility of hindered rotation of both the main chain and the side chains on the main chain, and other structural units. Thus the structural units on the polymer all contribute to the glass transition, as well as to stress relaxation. This can be characterized in terms of dynamic mechanical behavior by the loss modulus. This contribution of the groups to relaxation can be characterized by LA. It can complement the energy dissipation induced by the motion of whole chains and chain segments, i.e., physical damping. The contribution of the group to the loss modulus $G^{\prime \prime }$ is characterized by the loss area:

(15) $$\begin{array}{c} LA=\sum_{i=1}^{m}\frac{G_{i}}{M_{total}} \end{array}$$

where

(16) $$G_{i}=LA_{i}M_{i}$$

where $LA_{i}$ is the contribution of the *i*th molecular group to the area under the loss modulus curve of the material, $M_{i}$ is the relative molecular mass of the *i*th molecular group, $M_{total}$ is the total relative molecular mass of the polymer, $G_{i}$ is the molar loss of the *i*th molecular group, and *m* is the number of structural units in the polymer.

Chang et al. [35] synthesized the LA values and $G_{i}$ values of structural units of typical molecular chains. The contribution of some typical groups to the loss modulus was obtained, as shown in Table 1. In general, the larger the size of the group or the closer it is to the main chain, the more polar it is and the more it contributes to the *LA* value. However, when the length of the side group is very long, its effect is akin to that of a diluent, leading to a reduction in the *LA* value [35]. Similarly, the closer the group is to the main chain, the greater the contribution to the LA value. In this study, we refer to these data to quantitatively analyze the contribution of groups to the loss modulus, aiming to explain some of the mechanisms of material damping generation at the molecular level. Later, the calculated *LA* will be used for parameter identification of the model for group contribution based on the Kelvin–Voigt model.

### 2.3. Temperature Dependence of Mechanical Properties of Polymers

#### 2.3.1. Temperature Dependence of Viscoelastic Part

As the temperature increases, the viscosity of all branches decreases. This corresponds to a decrease in the relaxation time of each branch. For the GM model description of $M^{*}$, the relaxation time $\tau_{i}$ follows the time–temperature superposition principle (TTSP) [51,52]. The relaxation time at each temperature is related to the relaxation time at a fixed reference temperature as follows [53]:

(17) $$\tau_{i}\left(T\right)=\tau_{0}\alpha \left(T\right)$$

where $\alpha \left(T\right)$ is the temperature-dependent horizontal shift factor, and $\tau_{0}$ is the relaxation time of the reference temperature when the shift factor is 1.

For the temperature dependence of the relaxation time of the viscoelastic part, the shift factor can be obtained from the WLF equation [49,54] and the Arrhenius equation [27] at higher and lower temperatures, respectively (Figure 1b). It is generally assumed that the demarcation is near $T_{g}$, while in reality, there is no completely defined value for $T_{g}$ for polymers. So the demarcation can be determined from the $\alpha_{v}\left(T\right)-T$ figure. To ensure its continuity, the temperature of the demarcation is taken as the reference temperature $T_{r}$. The shift factor $\alpha \left(T\right)$ satisfies [53]:

(18) $$\begin{array}{c} \log_{10}\left[\alpha \left(T\right)\right]=\left\{\begin{array}{ccc} \; & 0.434\frac{H}{R}\left(\frac{1}{T}-\frac{1}{T_{r}}\right)\;, & \forall T<T_{r}\\ \; & -\frac{C_{1}\left(T-T_{r}\right)}{C_{2}+T-T_{r}}\;, & \forall T⩾T_{r} \end{array}\right. \end{array}$$

where the gas constant *R* is 8.31 J/(mol·K), *H* is the chain relaxation activation energy, and $C_{1}$ and $C_{2}$ are material parameters.

#### 2.3.2. Temperature Dependence of the Group Effect

The temperature dependence of the group effect is mainly reflected in the change in viscosity with temperature. The molecular theory of viscosity correlation is extremely complex at the molecular level. For smaller-scale groups, the free-volume theory can characterize the viscosity [55,56,57]: the overall polymer is integrated into the occupied volume and free volume. The free volume represents the fraction of voids in the polymer, which is the active space of the group when relaxation occurs. The larger the active space between the molecules, the less the motions of the groups are hindered, and the smaller the viscosity. According to the free-volume theory, the change in viscosity can be expressed by the free-volume fraction [57,58]:

(19) $$\log_{10}\left[\frac{\eta \left(T\right)}{\eta \left(T_{g}\right)}\right]=\frac{B}{2.303}\left(\frac{1}{f}-\frac{1}{f_{g}}\right)$$

where *B* is the material parameter, and *f* and $f_{g}$ are the free-volume fractions at *T* and $T_{g}$, respectively. The relationship between them can be derived from the specific volume–temperature curves of polymers, as shown in Figure 1c. The free volume is a constant until the glass transition is reached. The turn in the coefficient of thermal expansion at $T_{g}$ corresponds to the abrupt onset of free-volume expansion. Above $T_{g}$, the free-volume fraction can be expressed as

(20) $$f=f_{g}+\alpha_{f}\left(T-T_{g}\right)$$

where $\alpha_{f}$ is the coefficient of thermal expansion. Substituting it into Equation (19) gives the following relation:

(21) $$\log_{10}\left[\frac{\eta_{G}\left(T\right)}{\eta_{G}\left(T_{g}\right)}\right]=-\frac{B}{2.303}\left(\frac{T-T_{g}}{\frac{f_{g}}{\alpha_{f}}+T-T_{g}}\right)$$

This equation is another expression of the WLF equation. The experimental results show that the constant B is a value close to 1 for most polymers. From the WLF equation for the viscoelastic part, the free-volume fraction f and the free-volume expansion coefficient $\alpha_{f}\left(T_{g}\right)$ can be found.

So, for the viscosity of the part of group contribution, above $T_{g}$, the temperature dependence of the viscosity η(T) can be captured according to the WLF equation of the viscoelastic model. $C_{1}$ and $C_{2}$ of the viscoelastic part are taken as universal constants. Below $T_{g}$, the free-volume fraction can be approximated as a constant since the occupied volume of the polymer does not change much. The viscosity of the part of group contribution is

(22) $$\left\{\begin{array}{cc} \eta \left(T\right)=\eta \left(T_{g}\right)\;, & \forall T⩽T_{g}\\ \log_{10}\left[\frac{\eta \left(T\right)}{\eta \left(T_{g}\right)}\right]=-\frac{C_{1}\left(T-T_{g}\right)}{C_{2}+T-T_{g}}\;,\;\; & \forall T>T_{g} \end{array}\right.$$

where $C_{1}$ and $C_{2}$ are material parameters that can be calculated as universal constants for the viscoelastic part.

## 3. Experimental Section

### 3.1. Material Synthesis

The materials and processes for the preparation of the vitrimer of the disulfide bonds are as follows:

Detailed parameters of each experimental material can be found in Table 2. The normalized mole content of each composition was as follows: 1 epoxy group (BADGE) and 1 hardener (2-APD).

**Figure 3:**
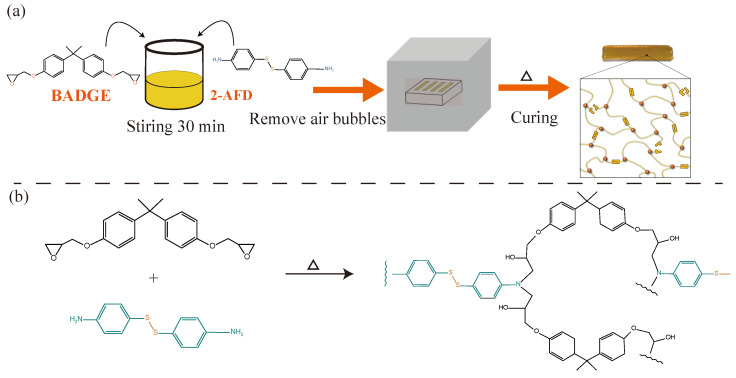
(**a**) Curing process of materials. (**b**) Synthesis and chemical structure of vitrimer of disulfide bonds.

The requisite amounts of BADGE and APD were mixed and mechanically stirred for 30 min (600 r/min) at 80 °C. Then, the mixture was degassed by vacuum to remove the air bubbles. Afterward, the solution was transferred to a PTFE mold using a syringe. The sample was obtained by curing the degassed mixture at 100 °C for 1 h, then at 130 °C for 1 h, and finally, at 150 °C for 5 h. The samples obtained contained an adaptive reversible crosslinked network composed of disulfide bonds [27]. Upon completion of the curing process, the crosslinked network was formed within the material. The preparation process of the material is shown in Figure 3.

### 3.2. Material Characterization

**Figure 4:**
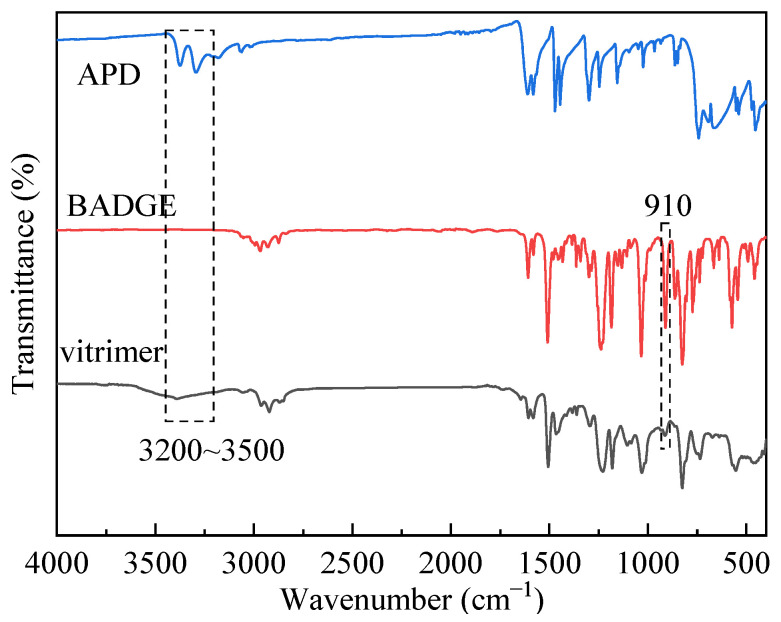
FT-IR spectra of APD, BADGE, and the vitrimer of disulfide bonds.

Fourier Transform Infrared Spectroscopy (FT-IR) was utilized to investigate the structure of the vitrimer of the disulfide bonds. Figure 4 presents the infrared spectra of the vitrimer of disulfide bonds, the epoxy resin monomer BADGE, and the hardener APD. Notably, the amino absorption peak of APD in the range of 3200–3500 cm^−1^ was not observed in the spectra of the vitrimer of the disulfide bonds. Additionally, the absorption peak corresponding to the epoxy group of BADGE at 914 cm^−1^ nearly vanished, indicating that the epoxy groups were largely consumed. These observations suggest that the reaction between the epoxy groups of BADGE and the amino groups of APD successfully facilitated the curing of BADGE, thereby providing evidence for the formation of a crosslinked network.

### 3.3. Dynamic Mechanical Analyzer

**Figure 5:**
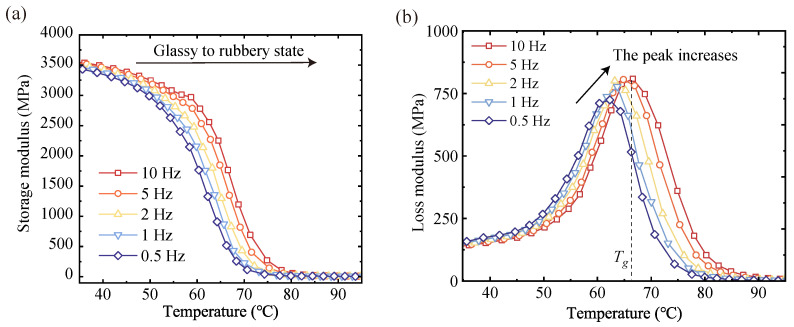
Temperature sweep results for DMA, including (**a**) storage modulus and (**b**) loss modulus.

Dynamic thermodynamic analysis (DMA) was performed on rectangular (45 × 10 × 4 mm) specimens of the vitrimer of the disulfide bonds to measure the storage modulus ($E^{\prime }$), loss modulus ($E^{\prime \prime }$), and damping loss factor (tanδ). The applied excitation frequencies were 0.5, 1, 2, 5, and 10 Hz. During the test, the temperature was increased from 20 °C to 130 °C at a rate of 2 °C/min. The instrument used for this DMA experiment was the Diamond DMA from PerkinElmer (Shanghai, China). The measurement mode was three-point bending, and a total of 4 samples were analyzed for this material. The experimental results are shown in Figure 5. The energy storage modulus and loss modulus were recorded. It can be observed that the energy storage modulus of the sample in the glassy state reaches 3.5 GPa. The storage modulus decreases rapidly after 50 °C and stabilizes after 85 °C. The modulus of the sample in the glassy state reaches 3.5 GPa. This region is taken as the glass transition region of the material. The modulus change in the polymer material is negligible in the glassy state at low temperatures and the viscous flow state region at high temperatures. The glass transition region between the two, and part of the glassy state region and the rubbery state region are taken as the main research objects. The temperature corresponding to the peak loss mode measured under 10 Hz excitation is taken as the glass transition temperature $T_{g}$ [59].

**Figure 6:**
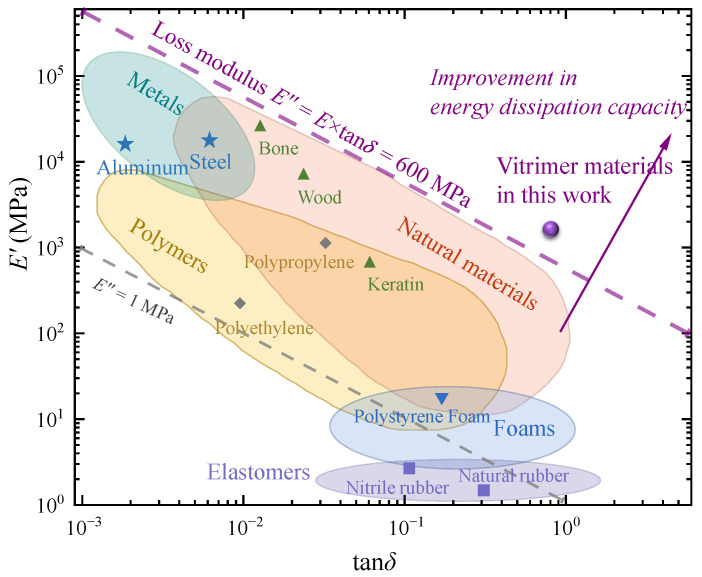
Stiffness–damping distribution of engineering and biomaterials [60,61].

In practical engineering applications, the loss modulus $E^{\prime \prime }$ is often utilized as a crucial parameter for assessing energy dissipation characteristics. Figure 6 shows the stiffness-damping distributions of various engineering and biological materials, with their eigenvalues typically falling below 600 MPa. Thanks to the pendant chains of the disulfide-crosslinked network, the material overcomes this limitation, indicating the potential existence of new mechanisms in the model characterization of the loss modulus.

## 4. Results and Discussion

### 4.1. Parameter Identification

#### 4.1.1. Parameters of the Viscoelastic Part

**Figure 7:**
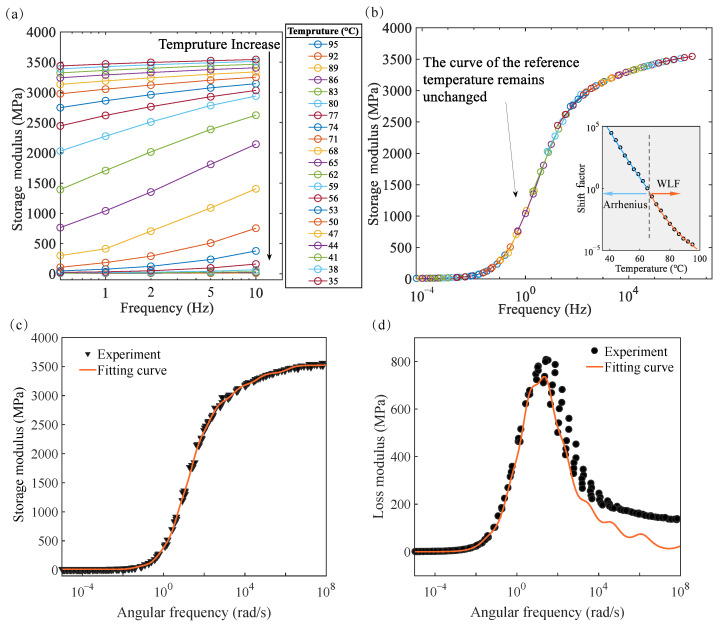
The process of obtaining master curves and parameter identification. (**a**) Storage modulus at different temperatures as a function of frequency. (**b**) Horizontal shift factor and fitting results for the viscoelastic part at each temperature. (**c**) Storage modulus master curve fitting results. (**d**) Loss modulus master curve fitting results.

A frequency response curve of the material was obtained to identify the model parameters. The computer programs of the parameter calibration method are all implemented in MATLAB 2024a. In the experimental data (Figure 5), it can be observed that the $E^{\prime }-T$ curves at different frequencies can overlap after shifting, but the $E^{\prime \prime }-T$ curves cannot. This means the loss modulus is not fully consistent with the TTSP and is difficult to simulate only through the GM model. Therefore the frequency domain-shifted results for the loss modulus have inevitable errors. Considering the $E^{\prime }-f$ curves as the variable to be shifted. The $E^{\prime }-f$ curves in the range of 35 to 95 °C were obtained by linear interpolation in steps of 3 °C (Figure 7a). The reference temperature $T_{r}$ was set at 65 °C, which is close to $T_{g}$.

According to the TTSP, the $E^{\prime }-f$ curve of each temperature is shifted to 65 °C, which is the storage modulus frequency master curve (Figure 7b). The shift factor for each temperature is recorded. The corresponding shift factor for 65 °C is 1. Based on the relationship between the shift factor and the temperature, the parameter is captured by Equation (18). The activation energy of chain relaxation is found to be 372.3 kJ/mol. $C_{1}$ and $C_{2}$ are 12.4 and 50.7 °C, respectively. The experimental data of the storage modulus and loss modulus are shifted according to the obtained shift factors. A frequency master curve of the dynamic response at $T_{r}$ is obtained. In this process, the universality of the TTSP is taken as a premise. However, since the loss modulus does not exactly match the TTSP, the storage modulus master curve, which has a higher confidence level, is used to identify the parameters. Group effects will be captured later in the $E^{\prime \prime }-f$ curve.

According to Equations (6), (9) and (12), the storage modulus and loss modulus as a function of angular frequency are

(23) $$\begin{array}{c} E^{\prime }\left(\omega \right)=\sum_{i=1}^{n}E_{i}\frac{\omega^{2}\tau_{i}^{2}}{1+\omega^{2}\tau_{i}^{2}}+E_{G}\\ E^{\prime \prime }\left(\omega \right)=i\sum_{i=1}^{n}E_{i}\frac{\omega \tau_{i}}{1+\omega^{2}\tau_{i}^{2}}+G^{\prime \prime } \end{array}$$

Due to the limitations of the Kelvin–Voigt model, $G^{\prime \prime }$ is not equal to $\omega \eta_{G}$ over the frequency domain. And because there are theoretical shift errors in the master curve of the loss modulus, it is more feasible to capture the parameters through the master curve of the storage modulus. The least minimum square method is used to calibrate the model parameters using the experimental data. When the number of Maxwell units is 1, it degenerates to a standard linear solid model. As shown in Figure S1, the modulus predicted by this model changes too quickly with frequency to describe the dynamic properties of the material. When *n* is gradually increased, the fit becomes gradually better and the curves become smoother. When n>9, the fitting effect of the model no longer changes. For this reason, we determined that n=9. The captured moduli and relaxation times are listed in Table 3. The results of the fit without $G^{\prime \prime }$ are shown in Figure 7b,c.

#### 4.1.2. Loss Modulus of the Group Effect

**Figure 8:**
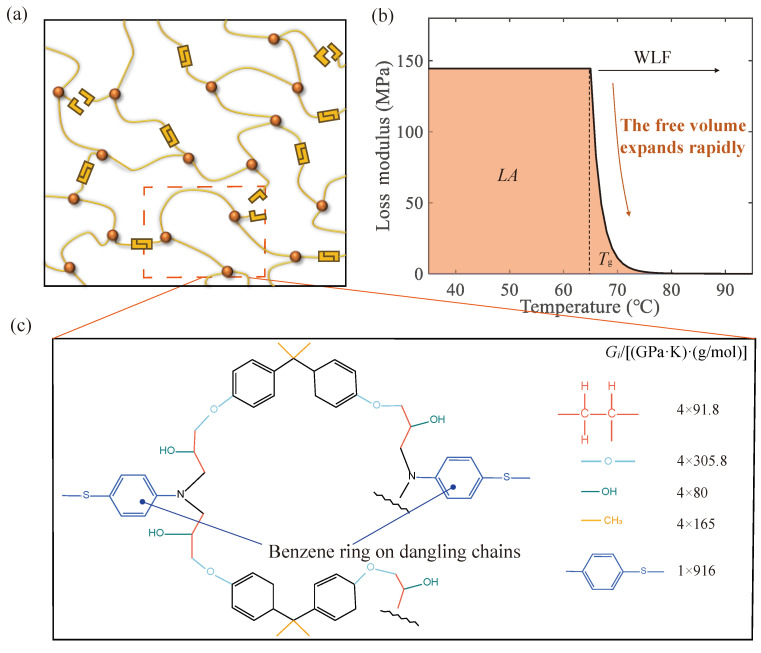
The loss modulus of the group effect. (**a**) A schematic diagram of the covalent adaptable network. (**b**) The calculated loss modulus of the group effect as a function of temperature. (**c**) Groups in the unit structure.

The vitrimer of the disulfide bonds is formed by combining a monomer of epoxy resin (BADGE) with a hardener (2-APD) in a 1:1 ratio. Therefore, it is structurally periodic. A representative unit structure was removed from the polymer, as shown in Figure 8c. The complex extended structure on the long side chains acts as a diluent equivalent at very long lengths. And since the polymer has a reticulated periodic structure, this contribution can be attributed to other unit chains without consideration. In contrast, dissipation from slipping between different network levels and between long chains can be considered to be a contribution on a larger scale. Therefore, only the contribution of motifs is considered here. According to Equation (15). Figure 8c shows the calculated data for group contribution. The LA obtained is 4.62 GPa·K.

In the frequency range of the experiment. The Kelvin–Voigt model is used to characterize the group-driven damping effect. From Equation (9), the loss modulus of the group effect can be expressed as

(24) $$G^{\prime \prime }\left(T\right)=\omega \eta_{G}\left(T\right)$$

The parameters are captured based on the LA obtained from the group contribution method. The loss modulus satisfies

(25) $$LA=\int_{T_{G}}^{T_{R}}G^{\prime \prime }\left(T\right)dT$$

where $T_{G}$ and $T_{R}$ are close to the starting and stopping temperatures of the glass transition behavior, respectively. In this instance, $T_{G}=35$ °C, $T_{R}=95$ °C. Based on the temperature dependence of the viscosity (Equation (22)), combined with Equations (24) and (25), the group-driven viscosity $\eta_{G}$ can be determined to be $2.3\times 10^{6}$ Pa·s. The relationship between the loss modulus and temperature near the glass transition region is shown in Figure 8b. The relevant parameters of the group part are shown in Table 4.

### 4.2. Comparison of Models and Experiments

The relaxation time for each temperature can be calculated based on the temperature dependence (Equation (17)). According to Equation (23), the $E^{\prime }-T$ curve and $E^{\prime \prime }-T$ curve can be obtained.

#### 4.2.1. Error in Viscoelastic Model without Group Effect

**Figure 9:**
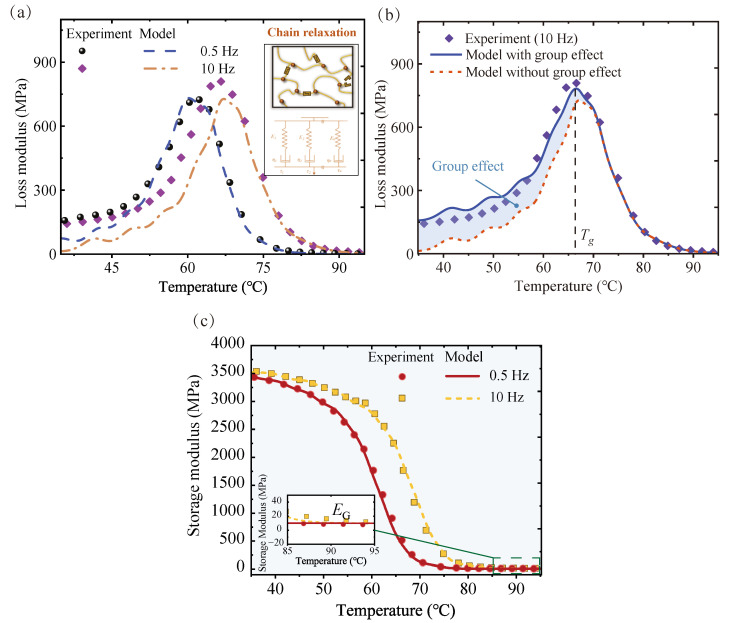
A comparison of the model with the experiment. (**a**) The loss modulus of the model without considering group effects. (**b**) Comparison of models with and without group effects. (**c**) A comparison of the storage modulus of the group-enriched model with the experiment.

Observing the comparison plots of the original model with the DMA experimental results (Figure 9a), it can be noticed that the temperature ranges in which there are large errors are different for the 0.5 Hz and 10 Hz models. At lower temperatures, the errors at both excitation frequencies are more pronounced. As the temperature increases, the model curve at 0.5 Hz gradually approaches the experimental results. And the model curve at 10 Hz gradually matches this after reaching the peak value. This gap originates from the different responses of the groups to different excitation frequencies. The essence of material viscoelasticity is that the movement of the internal structure under external excitation cannot fully keep up with the change in external force. At higher frequencies of external excitation, due to time constraints, the macromolecular chains do not have time to make conformational adjustments when the direction of the external force has already changed. In most cases, the molecular chains cannot keep up with the change in the direction of the stress. Groups are smaller moving units than chain segments. They have higher rotational freedom and lower rotational energy barriers. The motions of the groups drive the changes in bond angles and bond lengths, and the contribution to viscoelasticity gradually comes to the fore at high frequencies. Again, at lower temperatures, the chain segments in the polymer are essentially frozen. The viscoelastic effect arises mainly from smaller-scale motions, which are reflected in changes in bond angles and bond lengths. The motions of the groups provide the main driving force for this component.

#### 4.2.2. Predictive Performance of Group-Enriched Model

The comparison of group contributions (Figure 9b) reveals that the group-enriched viscoelastic model significantly reduces the discrepancy between the experimental loss modulus and the predicted loss modulus of the traditional viscoelastic model. The group effect primarily operates below the glass transition temperature $T_{g}$. The loss modulus attributed to the group effect diminishes the errors found in the conventional model at lower temperatures. Overall, the predictions of the loss modulus model align well with the experimental data in the range of 35–95 °C.

This analysis reveals that the contribution of the groups to the loss modulus is mainly in the glassy state and at the beginning of the glass transition zone. For the vitrimer of disulfide bonds, the higher degree of crosslinking leads to a shift in the glass transition zone towards higher temperatures. It has a higher operating temperature compared to conventional materials. At lower temperatures, the material is still in the glassy state. The rapid decay of the storage modulus has not yet occurred and the material still has good stiffness. At this time, the loss modulus of the material has not reached its peak, but it still has some energy dissipation capacity. This also plays a crucial role in broadening the application scenarios of the material.

The loss modulus is a performance parameter of interest for damping materials in freely damped structures. For most structures, the elastic modulus of the damping layer is two to three orders of magnitude smaller than that of the metallic structure [30]. In some cases, the elastic modulus in the glass transition region cannot easily satisfy this condition. So, the loss modulus of polymers in the glassy state is also of interest. The correction term of group contribution plays a key role in predicting the loss modulus at low temperatures. The importance of group movement is highlighted due to the freezing of the long chains at low temperatures. In this condition, the stiffness of the material is high, but the energy loss capacity is mediocre. As the temperature increases, the increase in loss modulus occurs simultaneously with the decrease in the storage modulus. By introducing a correction term to characterize the role of groups, the accuracy of the prediction of the loss modulus of the material can be improved, especially in the temperature interval where the stiffness is high. The predictions of the model can help to optimize the choice of operating temperature for polymers, thus improving the reliability of polymer materials.

For the storage modulus, the group-enriched model does not have an obvious advantage, but helps to explain the rubbery-state modulus mechanism. A comparison between the experiment and model $E^{\prime }-T$ curves is shown in Figure 9c.

As the glass transition gradually occurs, the mechanical properties of the material change dramatically, with the storage modulus rapidly decreasing by three to four orders of magnitude with increasing temperature. In this process, the free-volume fraction first remains essentially constant and rises rapidly at $T_{g}$. The alteration in the size of the group motion space contributes to the observed trend in the loss modulus (Figure 8b). Then, the polymer enters a rubbery state where the molecular chains move more freely. The damping effect of the material is substantially weakened, and the elastic deformation at the molecular level mainly provides the storage modulus. So, the storage modulus of the rubbery state is a molecular feature attributed to the contribution of groups.

**Figure 10:**
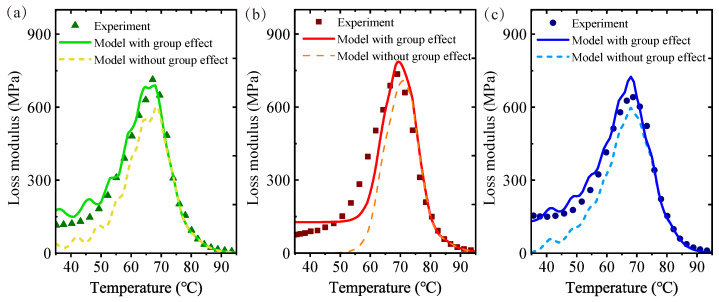
A comparison of the group-enriched model of the other three samples with experiments of 10 Hz: (**a**) Sample No. 1; (**b**) Sample No. 2; (**c**) Sample No. 3.

Additional samples of vitrimers with disulfide bonds synthesized from the same batch were investigated. To verify the validity of the group-enriched model, the dynamic mechanical properties of the other three samples were examined using the same approach, including DMA testing conducted under consistent conditions and the calibration of model parameters. The results of the experimental method, the group-enriched model (with the group effect), and the conventional viscoelastic model (without the group effect) are plotted in Figure 10. For the three samples, it is noteworthy that the group-enriched model is much closer to the experimental loss modulus. The group-enriched model compensates for the discrepancy between the conventional model and the experiment at low temperatures below $T_{g}$. This suggests that the group-enriched model has significant advantages over the conventional model.

The group-enriched model still has limitations. This model is studied with the excitation conditions of the DMA experiment. However, when the frequency is too high or too low, the simplified conditions for the model unit of the group contribution become difficult to fulfill, and the Kelvin–Voigt model is no longer applicable. The use of more complex phenomenology models may have better correction results, but also corresponds to more complex parameter identification methods.

## 5. Conclusions

A group-enriched viscoelastic model was developed for a damping material with many dangling chains where the group effect cannot be neglected. A specific damping material with many dangling chains was synthesized by using disulfide bonds, and its loss modulus surpasses the limited the loss modulus of conventional engineering materials. The group-enriched viscoelastic model can effectively improve the accuracy of the classical viscoelastic models and capture the experimental damping behavior of the synthesized damping material. Our results showed that the modified model can improve the prediction accuracy of the loss modulus. The freezing of chain segments at the beginning of the glassy and vitrification transitions leads to an increase in the influence of the groups on viscoelastic effects. The loss modulus is mainly provided by the viscosity of the motions of the groups. As the temperature increases, the chains and chain segments gradually thaw and the energy dissipation is dominated by chain relaxation. At the beginning of the glass transition and in the glassy state, the freezing of chain segments leads to an increase in the influence of the groups on the mechanical properties. The loss modulus is mainly influenced by the viscosity of the motions of the groups. As the temperature increases, the chains and chain segments gradually thaw and the energy dissipation is dominated by chain relaxation. When the temperature reaches $T_{g}$, free-volume expansion starts abruptly, and the space for molecular motion increases. The viscosity contributed by the groups decreases rapidly, and energy dissipation is dominated by chain relaxation. At low frequencies, the chain segments have sufficient time for extensive realignment, and the effect of the group can be ignored, whereas when the frequency is high enough, a flexibility gap between the chain segments and the motions of the group begins to manifest. The movement of the group in response to external excitation is more pronounced. Whereas the motion of the macromolecular chain cannot be kept in agreement with the external force, the group motion dominates the change in the loss modulus.

## Figures and Tables

**Table 1:** Contribution of different groups to *LA*.

Row	Group	Group Location	$G_{i}/\left[\left(\mathrm{GPa}\cdot K\right)\cdot \left(g/\mathrm{mol}\right)\right]$
1	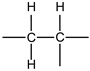	Backbone	91.8
2	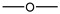	Backbone	305.8
3	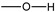	Side group	80
4	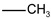	Side group	165
5	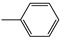	Side group	916

**Table 2:** Material parameters.

Materials	Item Model	Material Parameters	Manufacturer
2-Aminophenyl disulfide (2-APD)	A101816	Purity > 98%, 248.37 g/mol	Aladdin (Shanghai, China)
Bisphenol A diglicidyl ether (BADGE)	R007207	340.42 g/mol	RHAWN (Shanghai, China)

**Table 3:** The parameters of the viscoelastic part.

Types	Value	Description
$E_{i}$ (MPa)	920.6, 343.7, 1026.0, 133.2, 192.9, 76.5, 282.1, 66.5, 545.8	Modulus at 65 °C
$\tau_{i}$ (s)	2.35 $\times \;10^{-1}$, 1.76, 3.60 $\times \;10^{-2}$, 7.72 $\times \;10^{-7}$, 2.26 $\times \;10^{-5}$, 3.22 $\times \;10^{-9}$, 3.36 $\times \;10^{-4}$, 2.27 $\times \;10^{1}$, 4.59 $\times \;10^{-3}$	Relaxation time at 65 °C
*H* (kJ/mol)	372.3	Chain relaxation activation energy
$C_{1},C_{2}$ (°C)	12.4, 50.7	Constants of WLF equation
$E_{G}$ (MPa)	9.8	Storage modulus from group effect

**Table 4:** The parameters of the loss modulus of group contribution.

Parameter	Value	Description
$\eta \left(T_{g}\right)$ (Pa·s)	$2.3\times 10^{6}$	Viscosity of group contribution
LA (GPa·°C)	4.62	Loss area of group part
$T_{g}$ (°C)	66.6	Glass transition temperature determined by loss modulus

## Data Availability

The raw data supporting the conclusions of this article will be made available by the authors on request.

## Supplementary Materials

The following supporting information can be downloaded at: https://www.mdpi.com/article/10.3390/ma17205062/s1, Figure S1: The process of fitting the master curve in the frequency domain with the number of maxwell units *n* gradually increasing from 1 to 9.

## References

[1] Duan K., Li Y., Li L., Hu Y., Wang X. (2018). Pillared graphene as excellent reinforcement for polymer-based nanocomposites. Mater. Des..

[2] Shahsavari D., Karami B., Fahham H.R., Li L. (2018). On the shear buckling of porous nanoplates using a new size-dependent quasi-3D shear deformation theory. Acta Mech..

[3] Fan R., Meng G., Yang J., He C. (2009). Experimental study of the effect of viscoelastic damping materials on noise and vibration reduction within railway vehicles. J. Sound Vib..

[4] Wang F., Liao J., Huang C., Yu H., Yan J., Li H. (2022). Study on the damping dynamics characteristics of a viscoelastic damping material. Processes.

[5] Zhou X., Yu D., Shao X., Zhang S., Wang S. (2016). Research and applications of viscoelastic vibration damping materials: A review. Compos. Struct..

[6] Yang F., Sedaghati R., Esmailzadeh E. (2022). Vibration suppression of structures using tuned mass damper technology: A state-of-the-art review. J. Vib. Control.

[7] Treviso A., Van Genechten B., Mundo D., Tournour M. (2015). Damping in composite materials: Properties and models. Compos. Part Eng..

[8] Sujon M.A.S., Islam A., Nadimpalli V.K. (2021). Damping and sound absorption properties of polymer matrix composites: A review. Polym. Test..

[9] Huang Z., Qin Z., Chu F. (2014). A review about vibration problems of thin-walled structures with viscoelastic damping layer. J. Vib. Shock.

[10] Wang F., Li L., Tang H., Wang X., Hu Y. (2023). Damping of aluminum-matrix composite reinforced by carbon nanotube: Multiscale modeling and characteristics. Sci. China Technol. Sci..

[11] Mei C., Li L., Jiang Y., Ye Y., Li X., Han X., Tang H., Wang X., Hu Y. (2023). On band gap and damping of metamaterials involving negative-stiffness elements. Int. J. Mech. Sci..

[12] Woigk W., Poloni E., Grossman M., Bouville F., Masania K., Studart A.R. (2022). Nacre-like composites with superior specific damping performance. Proc. Natl. Acad. Sci. USA.

[13] Raquez J.M., Deléglise M., Lacrampe M.F., Krawczak P. (2010). Thermosetting (bio) materials derived from renewable resources: A critical review. Prog. Polym. Sci..

[14] Chatzimichali A.P., Potter K.D. (2015). From composite material technologies to composite products: A cross-sectorial reflection on technology transitions and production capability. Transl. Mater. Res..

[15] Wang S., Teng N., Dai J., Liu J., Cao L., Zhao W., Liu X. (2020). Taking advantages of intramolecular hydrogen bonding to prepare mechanically robust and catalyst-free vitrimer. Polymer.

[16] Xu C., Feng H., Li Y., Li L. (2024). Design of surpassing damping and modulus nanocomposites with tunable frequency range via hierarchical bio-architecture. Polym. Compos..

[17] Schenk V., Labastie K., Destarac M., Olivier P., Guerre M. (2022). Vitrimer composites: Current status and future challenges. Mater. Adv..

[18] Jiang Y., Li L., Hu Y. (2023). Strain gradient viscoelasticity theory of polymer networks. Int. J. Eng. Sci..

[19] Wu Y., Wei Y., Ji Y. (2023). Carbon material/vitrimer composites: Towards sustainable, functional, and high-performance crosslinked polymeric materials. Giant.

[20] Montarnal D., Capelot M., Tournilhac F., Leibler L. (2011). Silica-like malleable materials from permanent organic networks. Science.

[21] Brutman J.P., Delgado P.A., Hillmyer M.A. (2014). Polylactide vitrimers. Acs Macro Lett..

[22] Lyon G.B., Cox L.M., Goodrich J.T., Baranek A.D., Ding Y., Bowman C.N. (2016). Remoldable thiol–ene vitrimers for photopatterning and nanoimprint lithography. Macromolecules.

[23] Altuna F.I., Hoppe C.E., Williams R.J. (2019). Epoxy vitrimers with a covalently bonded tertiary amine as catalyst of the transesterification reaction. Eur. Polym. J..

[24] Ma Z., Wang Y., Zhu J., Yu J., Hu Z. (2017). Bio-based epoxy vitrimers: Reprocessibility, controllable shape memory, and degradability. J. Polym. Sci. Part Polym. Chem..

[25] Liu X., Zhang E., Feng Z., Liu J., Chen B., Liang L. (2021). Degradable bio-based epoxy vitrimers based on imine chemistry and their application in recyclable carbon fiber composites. J. Mater. Sci..

[26] Mai V.D., Shin S.R., Lee D.S., Kang I. (2019). Thermal healing, reshaping and ecofriendly recycling of epoxy resin crosslinked with Schiff base of vanillin and hexane-1, 6-diamine. Polymers.

[27] Krishnakumar B., Sanka R.S.P., Binder W.H., Park C., Jung J., Parthasarthy V., Rana S., Yun G.J. (2020). Catalyst free self-healable vitrimer/graphene oxide nanocomposites. Compos. Part Eng..

[28] Jawaid M., Khalil H.A., Hassan A., Dungani R., Hadiyane A. (2013). Effect of jute fibre loading on tensile and dynamic mechanical properties of oil palm epoxy composites. Compos. Part Eng..

[29] Unwin A.P., Hine P.J., Ward I.M., Fujita M., Tanaka E., Gusev A.A. (2018). Escaping the Ashby limit for mechanical damping/stiffness trade-off using a constrained high internal friction interfacial layer. Sci. Rep..

[30] Ball G.L., Salyer I.O. (1966). Development of a Viscoelastic Composition having Superior Vibration-Damping Capability. J. Acoust. Soc. Am..

[31] Lakes R.S. (2009). Viscoelastic Materials.

[32] Long R., Qi H.J., Dunn M.L. (2013). Modeling the mechanics of covalently adaptable polymer networks with temperature-dependent bond exchange reactions. Soft Matter.

[33] Ma J., Mu X., Bowman C.N., Sun Y., Dunn M.L., Qi H.J., Fang D. (2014). A photoviscoplastic model for photoactivated covalent adaptive networks. J. Mech. Phys. Solids.

[34] Luo C., Shi X., Lei Z., Zhu C., Zhang W., Yu K. (2018). Effects of bond exchange reactions and relaxation of polymer chains on the thermomechanical behaviors of covalent adaptable network polymers. Polymer.

[35] Chang M.C.O., Thomas D., Sperling L. (1988). Group contribution analysis of the damping behavior of homopolymers, statistical copolymers, and interpenetrating polymer networks based on acrylic, vinyl, and styrenic mers. J. Polym. Sci. Part Polym. Phys..

[36] Xu C., Li L. (2023). A surpassingly stiff yet lossy multiscale nanocomposite inspired by bio-architecture. Mater. Today Commun..

[37] Davis W., Szabo J.P. (2001). Group contribution analysis applied to the Havriliak–Negami model for polyurethanes. Comput. Theor. Polym. Sci..

[38] Li L., Lin R., Ng T.Y. (2020). A fractional nonlocal time-space viscoelasticity theory and its applications in structural dynamics. Appl. Math. Model..

[39] Jiang Y., Li L., Hu Y. (2023). A spatiotemporally-nonlocal continuum field theory of polymer networks. Sci. China Phys. Mech. Astron..

[40] Hatada T., Kobori T., Ishida M., Niwa N. (2000). Dynamic analysis of structures with Maxwell model. Earthq. Eng. Struct. Dyn..

[41] Renaud F., Dion J.L., Chevallier G., Tawfiq I., Lemaire R. (2011). A new identification method of viscoelastic behavior: Application to the generalized Maxwell model. Mech. Syst. Signal Process..

[42] Friedrich C. (1991). Relaxation and retardation functions of the Maxwell model with fractional derivatives. Rheol. Acta.

[43] Haghpanah B., Shirazi A., Salari-Sharif L., Izard A.G., Valdevit L. (2017). Elastic architected materials with extreme damping capacity. Extrem. Mech. Lett..

[44] Lin H., Xie S., Yong R., Chen Y., Du S. (2019). An empirical statistical constitutive relationship for rock joint shearing considering scale effect. Comptes Rendus. Mécanique.

[45] Kumar D., Lateefi M.M., Sarangi S. (2019). A phenomenological model for the viscoelastic behaviour of natural rubber. IOP Conference Series: Materials Science and Engineering.

[46] Besdo D., Ihlemann J. (2003). A phenomenological constitutive model for rubberlike materials and its numerical applications. Int. J. Plast..

[47] Fradkin D., Foster J., Sperling L., Thomas D. (1986). Molecular demixing in poly [cross-(ethyl acrylete)]-inter-poly [cross-(methyl methacrylate)] interpenetrating polymer networks brought about by selective decrosslinking and annealing. Polym. Eng. Sci..

[48] Fradkin D., Foster J., Sperling L., Thomas D. (1986). A quantitative determination of the damping behavior of acrylic based interpenetrating polymer networks. Rubber Chem. Technol..

[49] Fay J., Thomas D., Sperling L. (1991). Evaluation of the area under linear loss modulus-temperature curves. J. Appl. Polym. Sci..

[50] Sperling L., Fay J. (1991). Factors which affect the glass transition and damping capability of polymers. Polym. Adv. Technol..

[51] Yu K., Shi Q., Li H., Jabour J., Yang H., Dunn M.L., Wang T., Qi H.J. (2016). Interfacial welding of dynamic covalent network polymers. J. Mech. Phys. Solids.

[52] He X., Hanzon D.W., Yu K. (2018). Cyclic welding behavior of covalent adaptable network polymers. J. Polym. Sci. Part Polym. Phys..

[53] Rubinstein M., Colby R.H. (2003). Polymer Physics.

[54] Williams M.L., Landel R.F., Ferry J.D. (1955). The temperature dependence of relaxation mechanisms in amorphous polymers and other glass-forming liquids. J. Am. Chem. Soc..

[55] Duda J., Zielinski J.M. (1996). Free-volume theory. Plast. Eng. N. Y..

[56] Turnbull D., Cohen M.H. (1961). Free-volume model of the amorphous phase: Glass transition. J. Chem. Phys..

[57] White R.P., Lipson J.E. (2016). Polymer free volume and its connection to the glass transition. Macromolecules.

[58] Doolittle A.K. (1951). Studies in Newtonian flow. II. The dependence of the viscosity of liquids on free-space. J. Appl. Phys..

[59] Akay M. (1993). Aspects of dynamic mechanical analysis in polymeric composites. Compos. Sci. Technol..

[60] Ashby M.F. (1989). Overview No. 80: On the engineering properties of materials. Acta Metall..

[61] Qiu M., Wu S., Tang Z., Guo B. (2018). Exchangeable interfacial crosslinks towards mechanically robust elastomer/carbon nanotubes vitrimers. Compos. Sci. Technol..

