# Supplementary material for: A Group-Enriched Viscoelastic Model for High-Damping Vitrimers with Many Dangling Chains
Source: Materials (Basel). 2024 Oct 17;17(20):5062. doi: 10.3390/ma17205062 (PMC11509541; doi:10.3390/ma17205062)
Supplement: Supplementary file 1 [file materials-17-05062-s001.zip › materials-3200036-supplementary.pdf]

Supplementary Material for

Effects of Bond Exchange Reactions and Relaxation of Polymer Chains on the Thermomechanical Behaviors of Covalent Adaptable Network Polymers

Yan Li , Haibo Feng, Jing Xiong and Li Li*

State Key Laboratory of Intelligent Manufacturing Equipment and Technology, School of Mechanical Science and Engineering, Huazhong University of Science and Technology , Wuhan 430074, China

* Correspondence: lili_em@hust.edu.cn

1. Parameter identification process for the generalised Maxwell model

The fitting process of the generalized Maxwell model is illustrated in Figure S1. By gradually increasing the number of Maxwell units n, the model curve approaches the main curve in the frequency domain. Initially, we assume there is only one branch, i.e., $n = 1$. The start point for the modulus is set at 4000 MPa, which is close to the storage modulus at low temperatures. The start point for the relaxation time is set at 10 s. When there is only one unit, the model describes the modulus change too fast so it is difficult to describe the dynamic mechanical properties of the material. With each additional branch, the initial modulus for each branch is adjusted to $\frac{4000}{n}$ MPa, and the newly introduced relaxation time is set to be 0.1 times that of the previous branch. As n is gradually increased, the fit becomes gradually better and the curves become smoother.

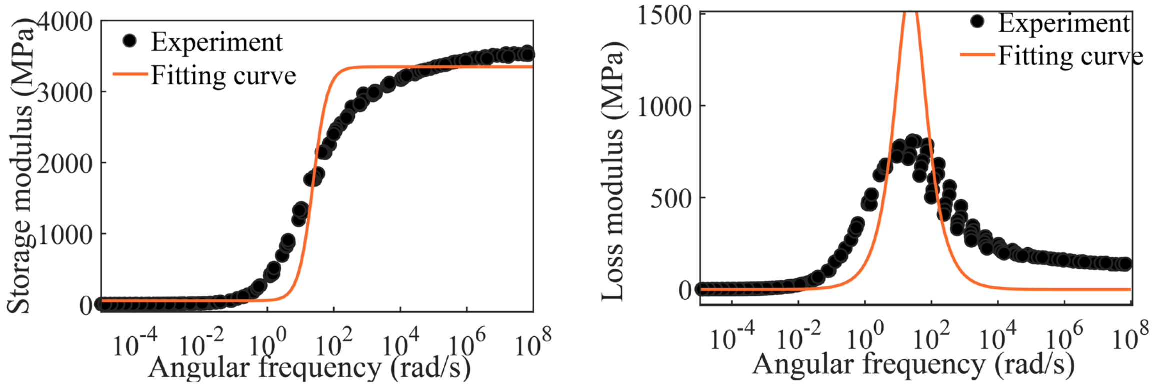

(a) Fitting results when the number of Maxwell units is 1

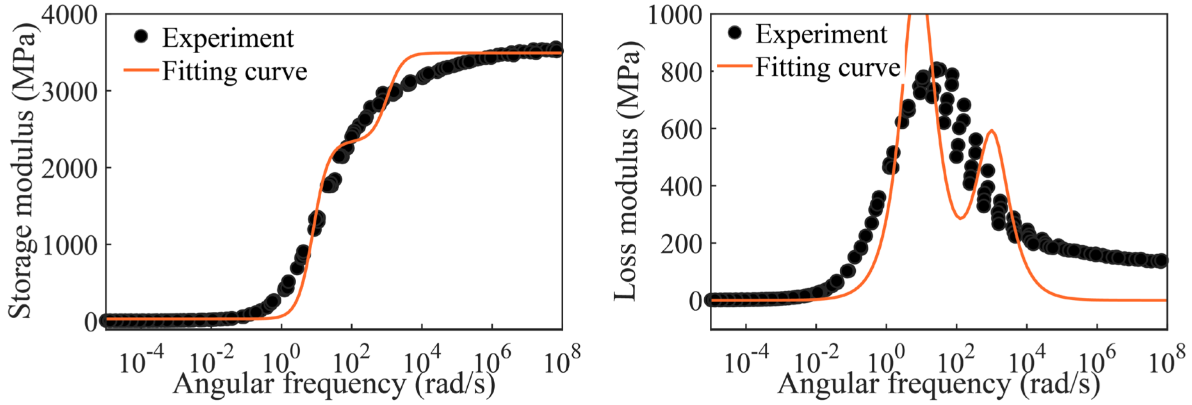

(b) Fitting results when the number of Maxwell units is 2

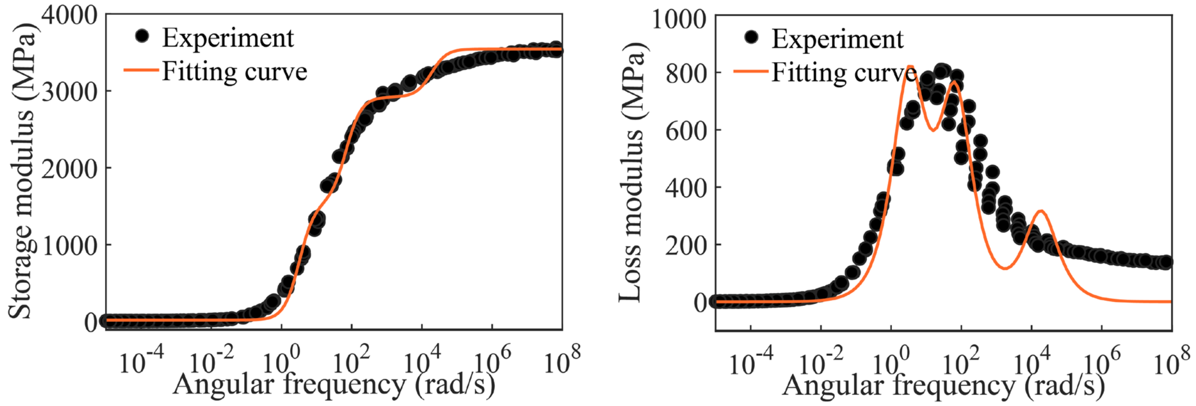

(c) Fitting results when the number of Maxwell units is 3

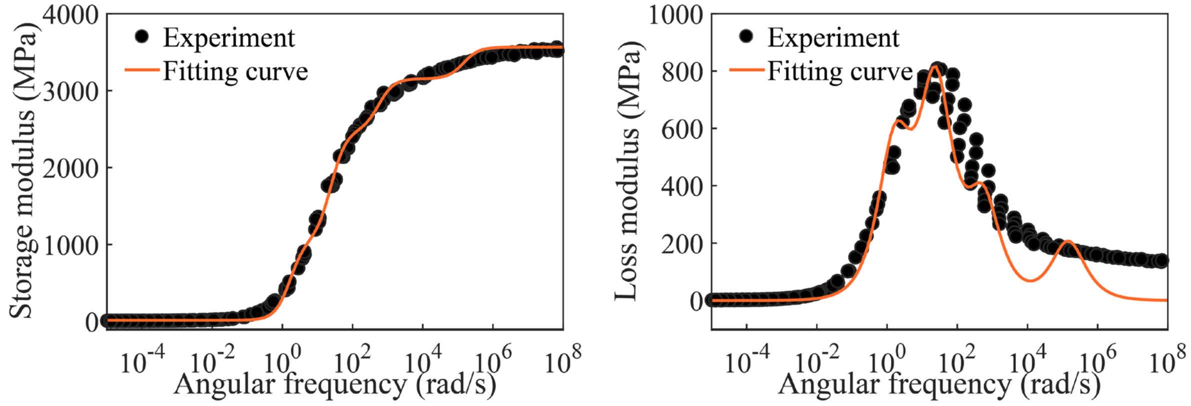

(d) Fitting results when the number of Maxwell units is 4

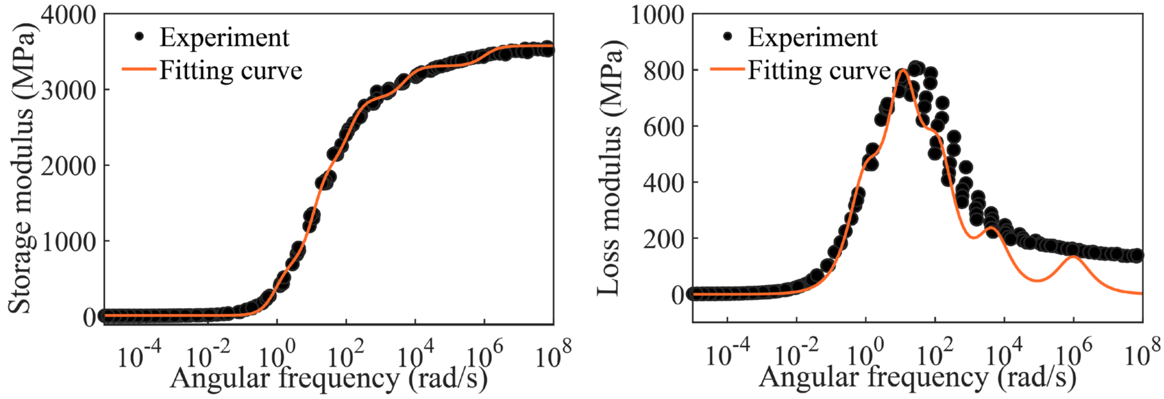

(e) Fitting results when the number of maxwell units is 5

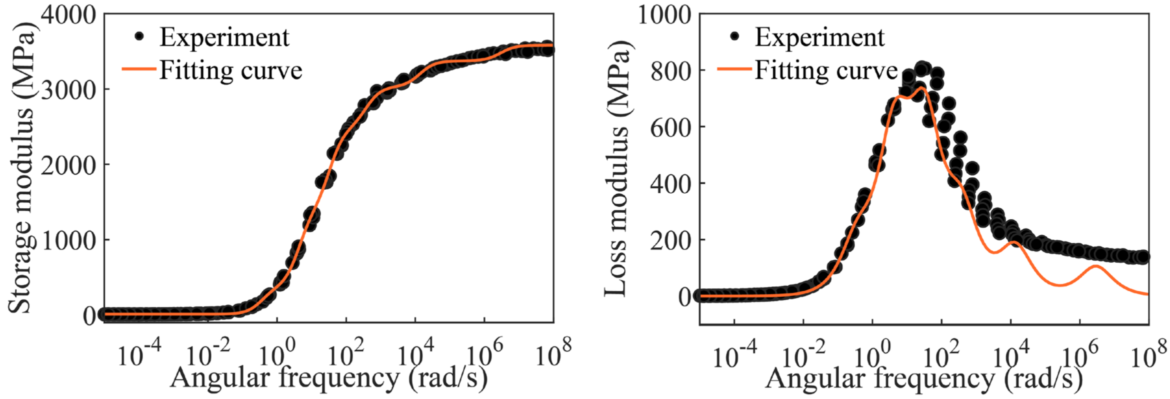

(f) Fitting results when the number of maxwell units is 6

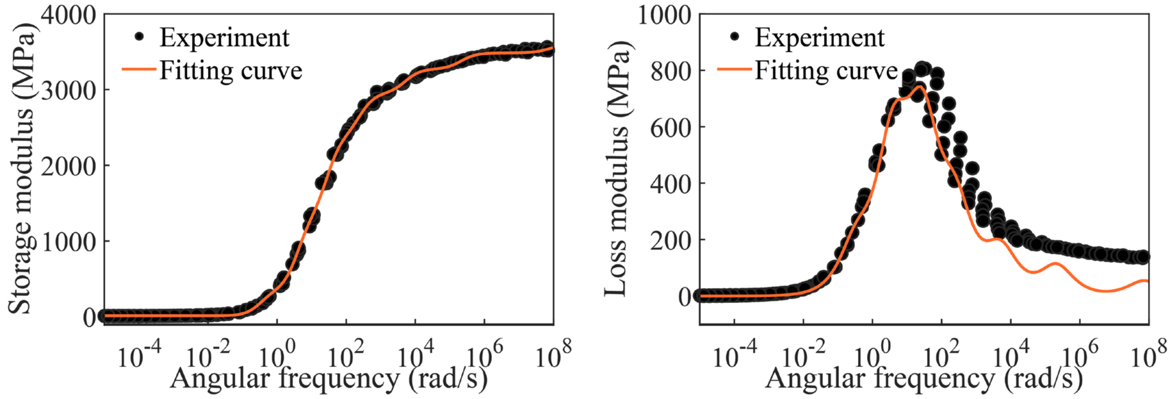

(g) Fitting results when the number of maxwell units is 7

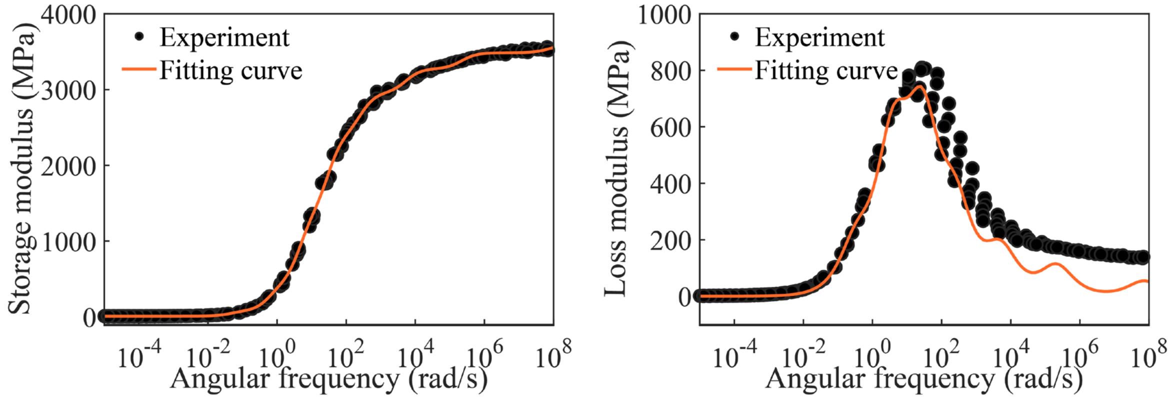

(h) Fitting results when the number of maxwell units is 8

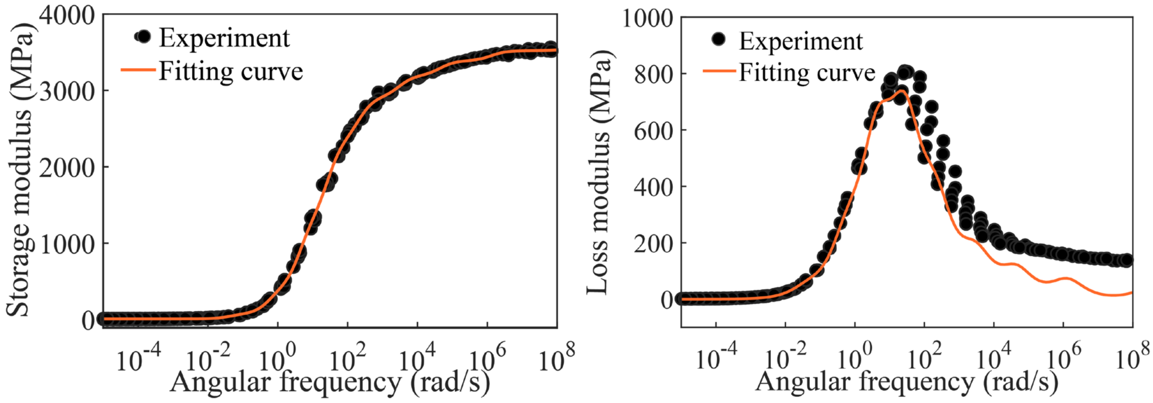

(i) Fitting results when the number of Maxwell units is 9

Figure S1 The process of fitting the master curve in the frequency domain with the number of maxwell units *n* gradually increasing from 1 to 9

2. Test Instrument Model

Fourier Transform Infrared Spectroscopy (FT-IR): Nicolet iS50R (Thermo Scientific).

DMA: Diamond (PerkinElmer).
